# Gender disparities and psychological distress among humanitarian migrants in Australia: a moderating role of migration pathway?

**DOI:** 10.1186/s13031-019-0196-y

**Published:** 2019-04-04

**Authors:** Yara Jarallah, Janeen Baxter

**Affiliations:** 1 Gender and Women’s Health, Centre for Health Equity, School of Population and Global Health, The University of Melbourne, Level 4, 207 Bouverie Street, Melbourne, VIC 3010 Australia; 2 ARC Centre of Excellence for Children and Families over the Life Course, Institute for Social Science Research, The University of Queensland Long Pocket Precinct, Indooroopilly, QLD 4068 Australia

**Keywords:** Gender, Mental health, Psychological distress, Refugees, Asylum seekers, Humanitarian migrants, Australia

## Abstract

**Background:**

The role of migration pathway (refugees vs. asylum seekers) is seldom addressed in extant literature that looks at gender and mental health of humanitarian migrants. The aim of this study is to assess the relationship between gender and psychological distress among humanitarian migrants in Australia including the potential moderating role of migration pathway.

**Methods:**

We analyse data from 2399 humanitarian migrants that participated in the first wave of Building a New Life in Australia, a survey of humanitarian migrants in Australia, using Ordinary Least Squares multivariate regression.

**Results:**

Women report significantly higher psychological distress than men. Migration pathway moderates the relationship between gender and psychological distress with women asylum seekers reporting higher psychological distress. There is also a significant association between pre-migration trauma, settlement arrangements (particularly those associated with finance, housing, getting used to life in Australia and loneliness) and psychological distress.

**Conclusions:**

Findings indicate higher psychological distress among asylum seeking women and suggest the importance of migrant status in predicting psychological distress. Settlement arrangements are key predictors of psychological distress among humanitarian migrants. While strategies aimed at addressing their mental health are warranted, policies aimed at the broader social determinants of health are needed to alleviate some of their mental distress especially in light of the recent changes to the Australian Refugee and Asylum-seeking policies.

**Electronic supplementary material:**

The online version of this article (10.1186/s13031-019-0196-y) contains supplementary material, which is available to authorized users.

## Background

Gender disparities in mental health has attracted a lot of international scholarship with decades of epidemiological and sociological research from both clinical and non-clinical population finding evidence of gender disparities in mental health [1, 2]. Incipient research extends this evidence base to humanitarian migrants [3–5], with refugee women being significantly more at risk of mental health conditions such as depression and Post Traumatic Stress Disorder- PTSD [6] and psychological distress [7] than refugee men. Few if any studies have looked at the potential role of migration pathway in moderating the association between gender and psychological distress- the aim of our current study.

Psychological distress can be defined as ‘the unpleasant subjective states of depression and anxiety, which have both emotional and physiological manifestations’ (p.8) [8]. It is an indicator of mental health that has been extensively used in population surveys, epidemiological studies and public health to assess variations in wellbeing [9]. Because exposure to stressful events that threaten physical and mental health are defining features of psychological distress [9] especially among humanitarian migrants- given the double stressors they endure before and after migration [9], it is used in this paper as a preferable indicator of mental health compared to more specific mental illnesses (for example, clinical depression and anxiety disorder). Indeed pre-migration experiences, especially exposure to traumatic events [10], and post-migration stresses associated with settling in a new country are significant predictors of psychological distress among humanitarian migrants [5, 11–13].

Humanitarian migrants include asylum seekers and refugees. Asylum seekers are people exercising their ‘right to seek protection under the 1951 UN Geneva Convention owing to a well-founded fear of being persecuted for reasons of race, religion, nationality, membership of a particular social group or political opinion’ (p.37) [14]. Refugees are people whose refugee status under this convention is already granted prior to their arrival in their host countries or countries of permanent settlement [14]. Few studies have looked at differences in mental distress between asylum seekers and refugees. The limited evidence to date suggests that asylum seekers fare the worst due to prolonged insecurity associated with their migrant status [4, 14, 15] and is especially pronounced among women [14]. The scarcity of evidence stems from lack of -until recently- nationally representative datasets on refugees and asylum seekers. In fact, amongst the studies cited here, only one uses a nationally representative sample from the population registers in the Netherlands [15]. The other studies use a convenience sample from immigrant drop-in centres, [14]and a selected sample of torture and trauma survivors [4]. Our study fills a gap by using a nationally representative data set of humanitarian migrants in Australia to investigate the relationship between gender and psychological distress. It is also the first- to the best of our knowledge- to examine whether humanitarian migrant type (i.e. migration pathway) moderates the relationship between gender and psychological distress. The entitlements and benefits associated with each migration pathway are different and might in turn have different impact on psychological wellbeing. Apart from our study, there are only two recent datasets that we know of that have comparable nationally representative data on humanitarian migrants; the UK Survey of New Refugees (2005–2009) and the German Socio-Economic Panel (SOEP) Sample of Refugees (2016–2018), but to date psychological distress has not been studied using these datasets.

The Australian context offers a unique opportunity to examine the psychological distress of humanitarian migrants in light of its built in program distinguishing refugees from asylum seekers. Formally established in 1977, the program consists of offshore and onshore components [16]. The former is for refugees and in country special humanitarian migrants who are mostly identified and referred to Australia by the United Nations High Commissioner for Refugees (UNHCR) to apply for a humanitarian visa outside Australia and are eligible for permanent residency and resettlement assistance [16, 17]. The latter is for asylum seekers who apply for protection after entering Australia with a temporary visa (i.e. a visitor or student visa) or without any documentation (also called ‘unauthorised arrivals’) [16].

The Australian policy towards humanitarian migrants and by extension, its humanitarian program has changed repeatedly since its inception in the late 1970s with the onshore program receiving the most frequent changes [16]. This started with the introduction of mandatory detention in 1992 for all onshore asylum seekers arriving in Australia ‘un-authorized’ with no documentation [16], a prolonged process that could take years until their refugee status was determined (p.1149) [4]. Notwithstanding concerns expressed by mental health professionals and others over the adverse psychological impact of prolonged detention on asylum seekers [4] including the scientific research substantiating those claims from cross-sectional [18, 19] and longitudinal studies [4, 20], Temporary Protection Visas (TPV) were subsequently introduced in 1999 for all asylum seekers who arrived in Australia ‘un-authorized’ but were later granted refugee status onshore [16]. This was the first time that Australia extended temporary protection provisions to the routine processing of individuals seeking asylum, forcing asylum seekers who have had their refugee status confirmed to re-submit their claims for protection every 3–5 years while confronting the possibility of future repatriation to their countries of origin (p.1150) [4]. Unlike humanitarian migrants whose refugee status is determined offshore, TPV holders are not granted the same federally funded benefits and services and up until 2008 were not eligible for full welfare assistance, English language tuition and family reunion sponsorship [4, 16, 21]. Although TPVs ceased in 2008 [22], they were reinstated in 2014 [16] and while their holders are allowed employment, Medicare, income support and English language tuition, there is no pathway for permanent residency nor family reunion provisions [23].

## Methods

### Data

This paper uses data from the first wave of Building a New Life in Australia (BNLA): The Longitudinal Survey of Humanitarian Migrants. BNLA is a longitudinal survey based on a representative sample of 2399 humanitarian migrants who arrived in Australia or were granted their permanent protection visa between May and December 2013 [24]. Face-to-face interviews with respondents was undertaken between October 2013 and March 2014 through home visits [24]. The study is funded by the Australian Department of Social Services (DSS) and administered by the Australian Institute of Family Studies (AIFS) [17]. The data is available publicly to authorized users through DSS. Both authors are authorized users and have received ethics exemption for using secondary data from the Office of Research Ethnics at the University of Queensland (clearance number: 2017001573).

### Study population

Selection into the study was based on migrating units (MUs) who had been granted permanent humanitarian visas 3–6 months preceding their first survey interview. The MU were identified through the Australian Government’s Department of Immigration and Border Protection Settlement Database and could consist of a single individual (principle applicant) or members of a family (principle applicant, secondary applicant adult and children). The study recruited participants from 11 Australian sites in all states and territories (except the Australian Capital Territory-ACT) across metropolitan and non-metropolitan areas [24]. Within those regions all permanent humanitarian visa holders were eligible. A total of 2399 MUs completed the survey representing 35 different countries of birth -the majority of which are from Iraq, Afghanistan and Iran- with about 50 different languages spoken at home and an overall response rate of 83% [24, 25]. This paper reports results from questions asked to both principle and secondary adult applicants 15 years and over and uses the corresponding population survey weights that adjust survey sample estimates to achieve population totals for all participants based on the Department of Immigration and Border Protection sampling frame [24].

### Measures

#### Psychological distress (K-6)

We measure psychological distress using the Kessler-6 Psychological Distress Scale (K-6)-a shorter version of the K-10 that performs just as well [26]. Originally designed for the US National Health Interview Survey, K-6 established validity and reliability in population based samples in the United States and internationally including Australia [27, 28]. Although validity has not been specifically established in refugee populations, it has been validated among different cultural groups including the Iraqi, Afghani and Iranian population to which most of our study population belongs to [29] and has also been used in refugee populations in Australia [29–31]. It has also been subsequently translated and validated cross-culturally across the five continents following its inclusion in the World Health Organization World Mental Health Survey Initiative without any considerable cultural bias [9, 26]. The K-6 consists of 6 questions that measure a person’s level of distress in the previous 4 weeks preceding the survey interview. The items included are: “How often did you feel… 1) Nervous?, 2) hopeless?, 3) restless/fidgety?, 4) that everything is an effort?, 5) so sad that nothing could cheer you up? and 6) worthless?”. Each item has five response categories “none of the time”, “a little of the time”, “some of the time”, “most of the time” and “all of the time” with a sum score range of 6–30 where higher scores indicate more psychological distress. The internal consistency reliability (Cronbach’s alpha) of the K-6 in the present study is 0.89.

#### Migration pathway

To be eligible for this study, permanent visa holders from the offshore pathway should have arrived in Australia 3–6 months prior to their first wave interview while permanent visa holders from the onshore pathway should have been granted their permanent protection visa 3–6 months prior to their first wave interview. Hence, unlike the offshore subgroup, onshore visa holders have been living in Australia for a longer time period either in immigration/community detention centres or on a different visa type [22]. The migration pathway indicator is a dichotomous variable with onshore and offshore categories which we label asylum seekers and refugees respectively (see Table 1).

**Table 1:** Sample and model variable characteristics (weighted)

	N	Percent
Psychological Distress^a^	2323	100
Gender		
Men	1296	54.0
Women	1103	46.0
Migration Pathway		
Refugee	1941	80.9
Asylum Seeker	458	19.1
Place of Birth		
MENA (exc. Iran & Iraq) ^b^	173	7.2
Iran	246	10.3
Iraq	778	32.4
Central & Southern Asia (exc. Afghanistan) ^c^	332	13.8
Afghanistan	482	20.1
Other ^d^	389	16.2
Age^e^	2399	100
Marital Status		
Married	1314	58.4
Separated/Divorced/Widowed	201	8.9
Never Married	733	32.6
Education		
Primary or Less	772	32.4
Preparatory	728	30.6
Secondary/Trade	588	24.7
University Degree	288	12.1
Previous Employment		
Yes	1252	52.6
No	1130	47.4
Previous Trauma Experience		
Yes	1975	82.3
No	424	17.7
Settlement Stressors		
Worry about family/friends overseas		
Yes	1221	52.9
No	1087	47.1
Getting used to life in Australia		
Yes	523	22.7
No	1785	77.3
Language Barriers		
Yes	1290	55.9
No	1018	44.1
Financial situation		
Yes	926	40.1
No	1382	59.9
House situation		
Yes	665	71.2
No	1643	28.8
Work situation		
Yes	760	32.9
No	1548	67.1
Loneliness		
Yes	366	15.9
No	1942	84.1
Family Safety		
Yes	368	16.0
No	1940	84.0
School/Study		
Yes	400	17.3
No	1908	82.7
Discrimination		
Yes	63	2.7
No	2245	97.3

^a^Mean = 12.83, SD = 5.82, Min = 6, Max = 30

^b^MENA includes Egypt, Libya, Sudan and Syria

^c^Central and Southern Asia includes Bhutan, India, Nepal, Pakistan and Sri Lanka

^d^Other includes Myanmar from Mainland South-East Asia, the Democratic Republic of Congo from Central and West Africa, and Eritrea and Ethiopia from Southern and East Africa

^e^Mean = 34.43, SD = 13.63, Min = 15, Max = 75

#### Socio-demographic measures

Our main predictor is gender measured as man or woman. Other socio-demographic measures include place of birth, age, marital status, education and previous employment (see Table 1). We also control for pre-migration trauma and post-migration stressors that have been shown in the literature to be associated with psychological distress among refugees [10–12]. Questions capturing pre-migration trauma are not from standard instruments (such as the Harvard Trauma Questionnaire) but capture similar questions about traumatic life events including extreme living conditions, war or other conflict, physical or sexual violence, imprisonment or kidnapping, political or religious persecution, natural disaster and other traumatic events. To save degrees of freedom and as the majority of the sample experienced some kind of trauma (82 precent), we create a dichotomous variable with yes for those who have experienced any trauma and no for those who have not (see Table 1). We also consider a categorical variable of cumulative trauma exposure with largely similar results (see Additional file 1: Table S1). Sources of stress are captured through a series of questions on several life domains (e.g. work, finance, housing, family) that respondents can separately select if the domain represents a source of stress and we only include stressors that are significantly associated with psychological distress at the bivariate level (see Table 1).

### Analysis

**Fig. 1:**
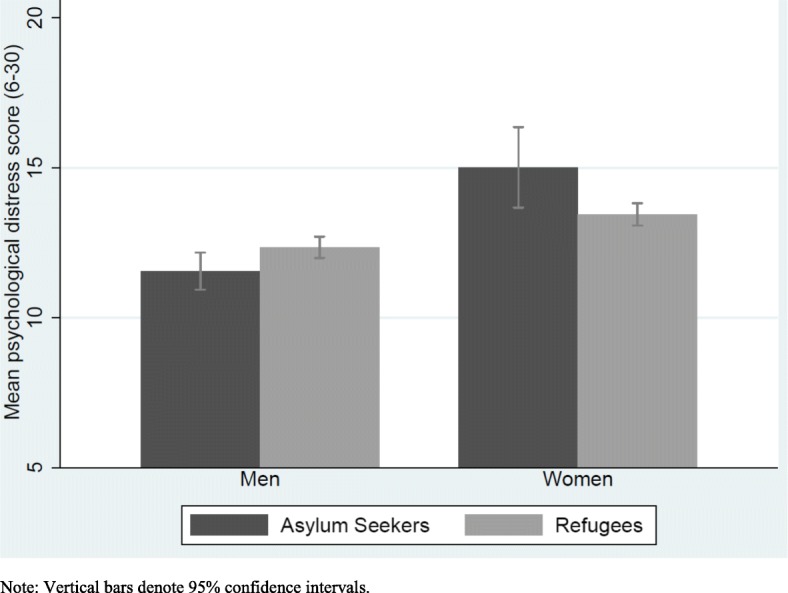
Psychological distress score by gender stratified by migration pathway, with 95% CI

Descriptive statistics are used to show the distribution of individual characteristics and model variables in the study sample (Table 1). Differences in psychological distress are depicted between men and women across the two migration pathways- refugees and asylum seekers (Fig. 1).

Subsequently, ordinary least squares (OLS) multivariate regression analysis is used to examine the association between gender, migration pathway and psychological distress (Table 2). We build our model step by step. Model I investigates the association between gender and psychological distress adjusted for socio-demographic variables. Model II tests the association between migration pathway and psychological distress above and beyond socio-demographic variables including gender. Model III investigates the influence of previous trauma experience and settlement stressors on the association between gender and psychological distress, adjusted for socio-demographics and migration pathway. Finally, (in model IV) we investigate whether migration pathway moderates the association between gender and psychological distress. All analyses are performed in Stata 14. Results are considered to be statistically significant at *p* < 0.05.

**Table 2:** OLS regression model of psychological distress (weighted)

	Model I	Model II	Model III	Model IV
	β	β	β	β
Gender				
Female	1.06***	1.03***	0.87**	2.34***
Male (ref.)				
Migration Pathway				
Refugee		0.31	0.56	1.08**
Asylum Seeker (ref.)				
Female*Refugee				−1.77**
Place of Birth				
MENA (exc. Iran and Iraq)	2.35***	2.50***	2.20***	2.01***
Iran	3.05***	3.11***	2.17***	2.14***
Iraq	3.17***	3.14***	2.60***	2.62***
Central & Southern Asia (exc. Afghanistan)	2.35***	2.41***	2.56***	2.61***
Afghanistan	0.75	0.80	0.39	0.43
Other (ref.)				
Age	0.03**	0.03**	0.02	0.02
Marital Status				
Married (ref.)				
Separated/Divorced/Widowed	1.62***	1.63**	0.96*	0.96*
Never Married	−0.45	−0.44	−0.22	−0.22
Education				
Primary or Less	−0.17	−0.27	−0.24	−0.20
Preparatory	−0.58	−0.66	−0.76	−0.75
Sector/Trade	−0.09	−0.15	−0.46	−0.46
University Degree (ref.)				
Previous Employment				
Yes (ref.)				
No	0.28	0.27	0.46	0.51
Previous Trauma Experience				
Yes			0.92**	0.97**
No (ref.)				
Settlement Stressors				
Worry about family/friends overseas				
Yes			0.03	0.07
No (ref.)				
Getting used to life in Australia				
Yes			0.70*	0.65*
No (ref.)				
Language Barriers				
Yes			0.26	0.25
No (ref.)				
Discrimination				
Yes			0.33	0.38
No (ref.)				
Financial situation				
Yes			1.64***	1.61***
No (ref.)				
House situation				
Yes			1.56***	1.54***
No (ref.)				
Work situation				
Yes			−0.42	−0.40
No (ref.)				
Loneliness				
Yes			2.41***	2.45***
No (ref.)				
Family Safety				
Yes			0.16	0.18
No (ref.)				
School/Study				
Yes			0.62	0.58
No (ref.)				
*N*	2155	2155	2107	2107
*R2*	0.0860	0.0864	0.1895	0.1924

*p < 0.05, **p < 0.01, ***p < 0.001

## Results

The study sample consisted of relatively more men (54%) than women and more refugees (81%) than asylum seekers. Respondents from the Middle East and North Africa (MENA) including Iran and Iraq comprise half the sample. The average age is 34 years, most are married (58%) and were employees or self-employed prior to migrating to Australia (53%). Thirty seven percent have secondary education or higher. Eighty two percent have suffered previous trauma and eighty nine percent have experienced at least one stressor since settling in Australia (Table 1).

Table 2 presents results from multivariate regression models that control for socio-demographic variables that may confound the relationship between gender, migration pathway and psychological distress. In Model I, adjusted for socio-demographic variables (place of birth, age, marital status, education, previous employment), women report higher psychological distress (β 1.06, *p* < 0.001) than men. In model II adjusted for socio-demographic variables and migration pathway, women report higher psychological distress (β 1.03, *p* < 0.001) than men but being a refugee is not significantly associated with psychological distress compared to being an asylum seeker. In the fully adjusted model (model III) including socio-demographic variables, migration pathway, pre migration trauma and post-migration stressors, women report higher psychological distress than men though the magnitude is reduced (β 0.87, *p* < 0.01).

Figure 1 shows the mean psychological distress score for men and women stratified by migration pathway. Regardless of migration pathway, mean psychological distress scores among women are higher than men. Looking within migration pathway, asylum seeking women have the highest psychological distress scores.

### Moderation by migration pathway

**Fig. 2:**
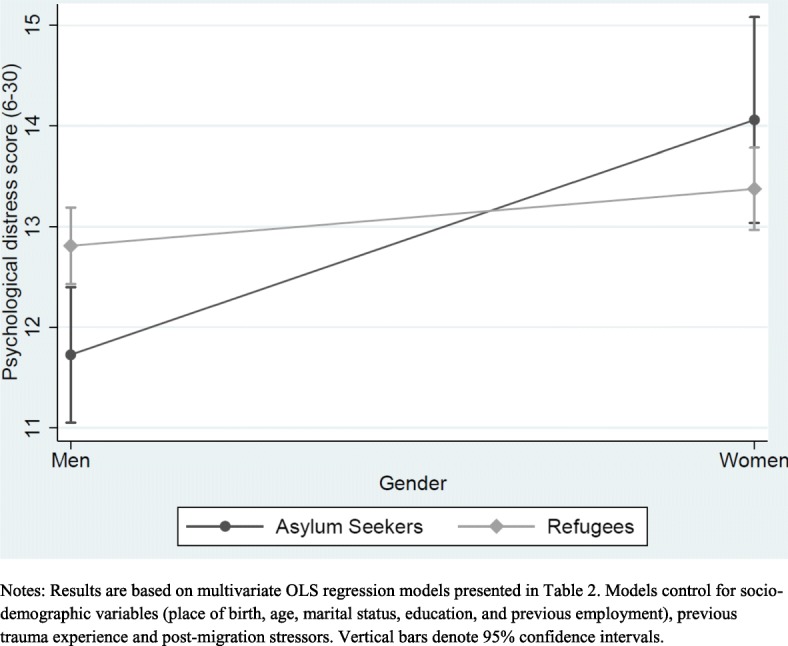
Interactions between gender and migration pathway

**Fig. 3:**
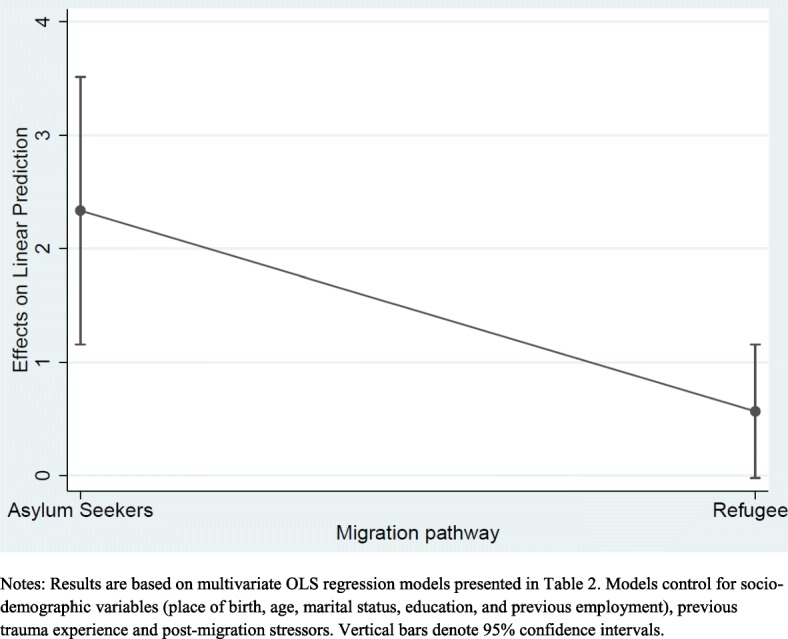
Marginal effects on psychological distress score of being woman in each migration pathway

To investigate whether migration pathway moderates the association between gender and psychological distress [Model IV from Table 2 reports the fully adjusted model with an interaction term between gender and migration pathway]. It is easier to interpret this relationship by inspecting Fig. 2. This shows that all else being equal, being a woman is associated with higher psychological distress than men and this is true for women in both migration pathways. Looking within migration pathway for women, being an asylum seeker is associated with higher psychological distress than a refugee. Although the interaction effect is significant in Table 2, the differences in psychological distress scores between women in each migration pathway may not be statistically significant as the confidence intervals overlap at the 95% level in Fig. 2. To investigate this further, Fig. 3 shows the marginal effect on psychological distress score for women in each migration pathway and suggests that the differences we observe are in fact statistically significant as denoted by non-overlapping confidence intervals at the 95% level.

Altogether, these results confirm the pattern observed in Fig. 1 and demonstrate statistically significant associations between gender and psychological distress with women and particularly asylum-seeking women showing the highest psychological distress.

### Pre-migration trauma and post-settlement stressors

In our sample (Table 2), all else being equal, persons who have experienced a trauma before migration to Australia report higher psychological distress than those who have not (β 0.97, *p* < 0.01). Among the post-settlement stressors that persons in our sample experience, and net of other variables, stressors from getting used to life in Australia (β 0.65, *p* < 0.05), their financial situation (β 1.61, *p* < 0.001), their housing situation (β 1.54, *p* < 0.001) and especially loneliness (β 2.45, *p* < 0.001) are significantly associated with psychological distress.

All else being equal, persons born in the Middle East and North Africa including Iran and Iraq and Central and Southern Asia including Afghanistan report higher psychological distress than persons born in other countries (Myanmar, Congo, Eritrea and Ethiopia) and with the exception of Afghanistan, these associations are statistically significant at (*p* < 0.001). Neither age nor education significantly predict psychological distress. Not being in paid employment prior to migrating to Australia is not significantly associated with psychological distress.

## Discussion

Our study is the first to examine the association between gender and psychological distress, as well as the moderating role of migration pathway using nationally representative Australian survey data from humanitarian migrants. Key findings show that women have higher psychological distress than men with women from the onshore (asylum seekers) pathway showing higher psychological distress than women from the offshore (refugees) pathway. These findings are consistent with the previous limited research from the USA [7], Ireland [14] and the Netherlands [15] but extend the evidence base of gender disparities in health among humanitarian migrants to Australia.

Extensive literature has been devoted to explain the gender gap in mental health particularly among those who have experienced previous trauma [32–34]. The type of trauma experienced has been shown to explain the gender gap [32]. In sensitivity analysis (results not shown), we indeed find evidence for this with women having significantly higher (*p* < 0.01) mean psychological distress scores on every type of trauma (except for natural disasters)- with traumas from conflict/war, physical or sexual violence and political or religious persecutions being very significant (*p* < 0.001). While the type of trauma experienced explains some of the differences, it does not fully explain away the gender gap [32, 33]. Sex differences in physiological and neurobiological stress response have also been suggested as potential mechanisms underlying the gender differences, although research advancement in this area is hampered by limited gender-sensitive research and reporting in the field of psychotraumatology and neurology [34, 35]. What is evident from this literature however, is a clear consensus that gender differences in mental health have a social underpinning linked to different gender roles and expectations [32–34, 36]. Indeed, the gendered pattern of social roles and social positions [2] is a result of differential access to power and prestige that produces inequalities in opportunities [8]. This means that female roles are more prone to competing social roles, role overload and mostly role limitations associated with lack of choice that puts female social positions in lower status levels (p.4) [2]. This might be amplified for humanitarian migrant women who have experienced both pre-migration trauma and post-settlement stresses in Australia that their sense of control over life might indeed by hindered. Control over one’s life has been shown to explain social patterns of distress [8]. Indeed, the broader social determinants of health associated with restricted economic opportunities, insecure housing, location of residence [37] and especially migration status can have a profound influence on one’s sense of control [4].

While the humanitarian migrant women in this study have all been granted permanent residency by the time they participated in the survey, those from the onshore pathway have either been living on temporary protection, visitor or student visas prior to being granted a permanent residency and have therefore been living in Australia longer than those from the offshore pathway who were granted permanent protection overseas. The higher psychological distress scores we observe for this group of humanitarian migrants might hence reflect longer exposure to settlement stressors. The uncertainty in migrant status associated with short-term visas with no associated federal benefits might also be another reason. While our current data does not allow us to unpack these effects-partly because the onshore group have only been on permanent protection for under a year, future research exploiting the longitudinal nature of the data as future waves become available is warranted. This can help determine whether the higher psychological distress we observe for the onshore humanitarian migrants is transient or long-lasting. Recent qualitative evidence on Afghani and Iraqi humanitarian migrants in Australia shows that psychological distress levels upon settlement persist over time and are higher for women [38]. While the study does not distinguish the onshore from offshore migrants-as our study does, it points to potential lasting impacts of past migratory experiences on psychological distress.

Consistent with previous research on humanitarian migrants, [10–12, 39] we find that those who have been exposed to trauma prior to migrating to Australia have higher psychological distress than those who have not. We also find that post-migration stressors are associated with psychological distress. In particular, financial stressors, housing stressors, loneliness, and getting used to life in Australia are all significant predictors of psychological distress. Our findings corroborate previous research documenting the effect of the post-migration stressors of finance, [14, 39] housing [5, 40] and loneliness [4, 39, 41] on psychological distress among humanitarian migrants [14, 39]. While housing stress is a growing concern in Australia with research evidence documenting its association with mental health and psychological distress, [42] its inclusion as a potential post-migration stressor has not been explored in research on humanitarian migrants in Australia. Our results reveal that it is a very significant predictor of psychological distress and hence should be included as a settlement stressor in future studies. Though tangential to our focus in this paper, the coefficients of place of birth from our control variables, offer insights into cultural differences associated with psychological distress that have been reported in the literature [43].

Our study has public health implications for humanitarian migrants in light of the significant association of post-migration stressors with psychological distress. While strategies aimed at addressing the mental health needs of humanitarian migrants are warranted, policies aimed at the broader social determinants of health are needed to alleviate some of the mental distress experienced by this population especially asylum seekers including those who arrived in Australia after 2014. As part of a series of changes to the Australian Refugee and Asylum Seeker policies, the TPVs, abolished in 2008, have been reinstated in 2014 [16, 22]. While visa holders can work and access publicly funded universal health care (Medicare), it is temporary with no family reunion provision nor a pathway for permanent residency [23]. Though our study sample has not been affected by this new policy, our results of significant psychological distress among asylum seekers and particularly women, is indicative for future immigrants under this scheme. In fact, it may be that our results underestimate the impact of settlement policies on mental health given less stringent policies prior to 2014. Further, the most recent changes in April 2017, has further implications for permanent visa holders (from both the onshore and offshore programs) in our sample who have not yet been granted their Australian citizenship especially considering evidence of considerable delays experienced by refugees when applying for citizenship [23]. Since April 2017, criteria for Australian citizenship has changed to include the following: having to be a permanent resident for 4 years, having to acquire close to a university entry level score on an English test, an additional module to assess people’s understanding of Australian values in the citizenship test and capping the number of times a person can re-take the test to three times, all of which, especially the English language requirements, are believed to disadvantage refugees [23].

There are some caveats to our study. First, while evidence did show that women from the onshore pathway have higher psychological distress, the sample is too small for analysis running separate models by migration pathways. Second, our data is cross-sectional and so causation cannot be implied and our results remain associational. Future research using subsequent waves as they become available can assess whether the associations observed here persist overtime. Nevertheless, our study provides valuable baseline information as the first to document gender disparities among a nationally representative sample of humanitarian migrants in Australia.

## Conclusions

In this study we extend the evidence on gender disparities in mental health to humanitarian migrants in Australia. Using a nationally representative sample of humanitarian migrants, we show the important role of migration pathway in mediating the relationship between gender and psychological distress. Our study also finds that post-migration stressors, particularly, financial and housing stressors, loneliness and getting used to life in Australia, are significantly associated with psychological distress. As such, strategies aimed at addressing the mental health needs of humanitarian migrants are warranted, but also policies aimed at the broader social determinants of health are much needed to alleviate some of the mental distress experienced by humanitarian migrants, particularly asylum seekers.

## Additional file

Additional file 1: **Table S1.** OLS regression model of psychological distress (weighted). (DOCX 15 kb)
