# Supplementary material for: Gender disparities and psychological distress among humanitarian migrants in Australia: a moderating role of migration pathway?
Source: Confl Health. 2019 Apr 4;13:13. doi: 10.1186/s13031-019-0196-y (PMC6449952; doi:10.1186/s13031-019-0196-y)
Supplement: Supplementary file 1 — Table S1. OLS regression model of psychological distress (weighted). (DOCX 15 kb) [file 13031_2019_196_MOESM1_ESM.docx]

**Additional file 1: Table S1. OLS regression model of psychological distress (weighted)**

|  | Model III | Model IV |
| --- | --- | --- |
|  | β | β |
| Gender |  |  |
| Female | 0.86** | 2.29*** |
| Male (ref.) |  |  |
| Migration Pathway |  |  |
| Refugee | 0.45 | 0.97* |
| Asylum Seeker (ref.) |  |  |
| Female*Refugee |  | -1.73** |
| Place of Birth |  |  |
| MENA (exc. Iran and Iraq) | 2.11*** | 1.92** |
| Iran | 2.08*** | 2.04*** |
| Iraq | 2.79*** | 2.81*** |
| Central & Southern Asia (exc. Afghanistan) | 2.50*** | 2.55*** |
| Afghanistan | 0.42 | 0.46 |
| Other (ref.) |  |  |
| Age | 0.03* | 0.02* |
| Marital Status |  |  |
| Married (ref.) |  |  |
| Separated/Divorced/Widowed | 0.97* | 0.97* |
| Never Married | -0.13 | -0.14 |
| Education |  |  |
| Primary or Less | -0.12 | -0.08 |
| Preparatory | -0.62 | -0.61 |
| Sector/Trade | -0.38 | -0.38 |
| University Degree (ref.) |  |  |
| Previous Employment |  |  |
| Yes (ref.) |  |  |
| No | 0.42 | 0.46 |
| Cumulative Previous Trauma Experience |  |  |
| 0 (ref.) |  |  |
| 1 | 1.41*** | 1.46*** |
| 2 | 0.79 | 0.85* |
| 3 or more | 0.10 | 0.16 |
| Settlement Stressors |  |  |
| Worry about family/friends overseas |  |  |
| Yes | 0.15 | 0.19 |
| No (ref.) |  |  |
| Getting used to life in Australia |  |  |
| Yes | 0.69* | 0.64* |
| No (ref.) |  |  |
| Language Barriers |  |  |
| Yes | 0.33 | 0.33 |
| No (ref.) |  |  |
| Discrimination |  |  |
| Yes | 0.46 | 0.50 |
| No (ref.) |  |  |
| Financial situation |  |  |
| Yes | 1.71*** | 1.68*** |
| No (ref.) |  |  |
| House situation |  |  |
| Yes | 1.58*** | 1.56*** |
| No (ref.) |  |  |
| Work situation |  |  |
| Yes | -0.41 | -0.39 |
| No (ref.) |  |  |
| Loneliness |  |  |
| Yes | 2.49*** | 2.52*** |
| No (ref.) |  |  |
| Family Safety |  |  |
| Yes | 0.29 | 0.31 |
| No (ref.) |  |  |
| School/Study |  |  |
| Yes | 0.60 | 0.56 |
| No (ref.) |  |  |
| *N* | 2107 | 2107 |
| *R2* | 0.1963 | 0.1991 |

*p<0.05, **p<0.01, ***p<0.001
